# *Trans* ε-Viniferin Decreases Amyloid Deposits With Greater Efficiency Than Resveratrol in an Alzheimer’s Mouse Model

**DOI:** 10.3389/fnins.2021.803927

**Published:** 2022-01-06

**Authors:** Aline Freyssin, Agnès Rioux Bilan, Bernard Fauconneau, Laurent Galineau, Sophie Serrière, Clovis Tauber, Flavie Perrin, Jérôme Guillard, Sylvie Chalon, Guylène Page

**Affiliations:** ^1^EA3808 Neurovascular Unit and Cognitive Disorders, University of Poitiers, Poitiers, France; ^2^UMR 1253, iBrain, Inserm, Faculty of Medicine, Université de Tours, Tours, France; ^3^UMR CNRS 7285 IC2MP, Team 5 Organic Synthesis, University of Poitiers, Poitiers, France

**Keywords:** Alzheimer’s disease, viniferin, resveratrol, PET imaging, amyloid deposits, memory decline

## Abstract

In a previous study, we showed that viniferin decreased amyloid deposits and reduced neuroinflammation in APPswePS1dE9 transgenic mice between 3 and 6 months of age. In the present study, wild type and APPswePS1dE9 transgenic mice were treated from 7 to 11 or from 3 to 12 months by a weekly intraperitoneal injection of either 20 mg/kg viniferin or resveratrol or their vehicle, the polyethylene glycol 200 (PEG 200). The cognitive status of the mice was evaluated by the Morris water maze test. Then, amyloid burden and neuroinflammation were quantified by western-blot, Enzyme-Linked ImmunoSorbent Assay (ELISA), immunofluorescence, and *in vivo* micro-Positon Emission Tomography (PET) imaging. Viniferin decreased hippocampal amyloid load and deposits with greater efficiency than resveratrol, and both treatments partially prevented the cognitive decline. Furthermore, a significant decrease in brain uptake of the TSPO PET tracer [^18^F]DPA-714 was observed with viniferin compared to resveratrol. Expression of GFAP, IBA1, and IL-1β were decreased by viniferin but PEG 200, which was very recently shown to be a neuroinflammatory inducer, masked the neuroprotective power of viniferin.

## Introduction

Alzheimer’s disease (AD) is the most common neurodegenerative disorder and the first cause of dementia in the world. Each year, there are over 9.9 million new cases of dementia worldwide, implying one new case every 3 s (Prince, 2015). Thus, in the world, nearly 50 million people have Alzheimer’s or related dementia as published by the Alzheimer’s association in 2021. This number will almost double every 20 years, reaching 75 million in 2030 and more than 130 million in 2050 (Prince, 2015). Unfortunately, there are no effective treatments to cure AD.

The cause of this disease is unknown, but many pathogenic mechanisms induce several types of cellular and molecular damage, e.g., extracellular senile plaques, composed of aggregated amyloid beta (Aβ) peptide, the intracellular neurofibrillary tangles composed of hyperphosphorylated tau protein, the exacerbated neuroinflammation, the synaptic loss in particular cholinergic hippocampal neurons and the oxidative stress (Tönnies and Trushina, 2017; Long and Holtzman, 2019; Webers et al., 2020). The pharmacological drugs approved by the authorities and currently used are acetylcholinesterase inhibitors (Singh and Sadiq, 2021) and uncompetitive antagonists of NMDA receptors (Alam et al., 2017), but these treatments are only symptomatic and do not prevent or cure the pathological mechanisms involved in AD and neuronal death (Mangialasche et al., 2010; Anand et al., 2014). Moreover, anti-amyloid-β and tau immunotherapies in clinical trials for AD have failed due to their late administration in the disease progression (Pedersen and Sigurdsson, 2015; van Dyck, 2018). Clinical trials evaluating the effects of drugs targeting only one pathological aspect of AD, such as neuroinflammation (Ali et al., 2019) or oxidative stress (Mecocci and Polidori, 2012; Charemboon and Jaisin, 2015; Wojsiat et al., 2018), failed to demonstrate the efficiency of these treatments. In this context, pharmacological treatments targeting several characteristic lesions of AD simultaneously could be more efficient. Consequently, natural polyphenols, which have been reported to possess various biological properties, could be interesting candidates (Dhakal et al., 2019). Polyphenols have been found to have anti-inflammatory action (Capiralla et al., 2012; Caillaud et al., 2019; Román et al., 2019), inhibit Aβ aggregation and induce its disaggregation (Ladiwala et al., 2010; Freyssin et al., 2018; Vion et al., 2018), inhibit phosphorylation and aggregation of tau and have protective effects against Aβ-induced neuronal death (Bureau et al., 2008; Vion et al., 2018; Sureda et al., 2019). One of them, *trans*-resveratrol (4-[(*E*)-2-(3,5-Dimethoxyphenyl)ethenyl]phenol) has been widely described in the literature for its beneficial properties on health, notably in the context of neurodegenerative diseases (Kou and Chen, 2017; Sawda et al., 2017; Chen et al., 2019). However, only one clinical trial on the beneficial effects of this stilbene has been reported in two papers. The first article found that resveratrol, administered to mild to moderate AD patients was safe and well tolerated but had ambivalent effects (Turner et al., 2015) in that resveratrol treatment compared to *placebo* induced a beneficial inhibition of Aβ decrease in cerebrospinal fluid (CSF) but also, an increase of in brain volume loss. Moreover, this treatment was reported to modulate neuroinflammation, and induce adaptive immunity (Moussa et al., 2017). Although better performances in cognitive tasks were observed in resveratrol-treated AD mice (Chen et al., 2019), clinical trials failed to show any efficacy on cognitive functions (Kennedy et al., 2010; Drygalski et al., 2018).

Resveratrol is rapidly metabolized in glucuronidated and sulfated forms which are excreted in the urine. Consequently, high doses are required but also induce adverse effects (Turner et al., 2015). Another natural polyphenol, *trans* ε-viniferin (5- [(2*R*,3*R*)-6-Hydroxy-2-(4-hydroxyphenyl)-4-[(*E*)-2-(4-hydroxyphenyl) ethenyl]-2,3-dihydro-1-benzofuran-3-yl]benzene-1,3-diol) recently caught our attention. It is a dimer of *trans*-resveratrol, synthetized by *Vitis vinifera* in response to different stresses and found in grapevine canes and roots. Viniferin could have higher beneficial properties than the reference polyphenol resveratrol, due to its chemical structure and reduced catabolism (Boocock et al., 2007). However, only a few findings concerning its role in AD have been published (Jeong et al., 2010; Rivière et al., 2010; Richard et al., 2011, 2013; Vion et al., 2018; Caillaud et al., 2019). In a first *in vitro* study, we confirmed that viniferin inhibited amyloid aggregation but we also demonstrated that it induced the disaggregation of amyloid fibrils with higher efficiency than resveratrol (Vion et al., 2018). Moreover, we showed that this stilbene rescued gliosis induced by aggregated Aβ in a murine primary culture of neurons, astrocytes and microglia, with better efficacy than resveratrol (Vion et al., 2018). In a second *in vivo* study, using APPswePS1dE9 transgenic mice, we evaluated the preventive effect of viniferin (Caillaud et al., 2019). AD mice received a weekly intraperitoneal (i.p.) injection of viniferin 10 mg/kg from 3 until 6 months. We reported that viniferin was able to pass through the blood brain barrier and had beneficial preventive effects, in that it reduced the size and density of amyloid deposits and decreased the reactivity of astrocytes and microglia (Caillaud et al., 2019). Therefore, as in these mice the characteristic histopathological lesions of the disease are not yet present at 3 months of age and appear from 4 to 6 months, this study only demonstrated the preventive effects of viniferin. The effects of treatment after development of the characteristic histopathological lesions, i.e., later than 6 months of age, remain unknown. Moreover, these beneficial effects were not compared to those of resveratrol. Lastly, it is would be necessary to determine whether these polyphenols could rescue the cognitive decline. For all these reasons, we decided to administer resveratrol or viniferin from 7 to 11 months or from 3 to 12 months in APPswePS1dE9 and WT mice to evaluate the efficacy of a chronic treatment. The aim of this study was therefore to compare the effects of the two polyphenols on (1) amyloid deposits, (2) neuroinflammation, and (3) cognitive decline. We previously demonstrated that a concomitant increase in the β-amyloid load and index of microglial activation 18 kDa translocator protein (TSPO) was detected in this Tg model by micro-positon emission tomography (PET) imaging (Sérrière et al., 2015).

## Materials and Methods

### Chemical Products

All chemical products used in this study are indicated in the Supplementary Table 1.

### Animals and Experimental Design

In the laboratory, we have APPswePS1dE9 transgenic mice (from Mutant Mouse Resource and Research Center, Stock No: 34829-JAX) displaying Alzheimer phenotype [Authorization from “Haut Comité de Biotechnologie français” (HCB) to Pr Guylène Page, number 2040 for reproduction, treatment, behavioral tests, and *ex vivo* experiments]. Wild type (WT) with B6C3F1 background and APPswePS1dE9 mice were obtained by crossing a male APPswePS1dE9 mouse with a WT female mouse (from Charles River, strain Code 031). The use of animals was approved by the Ethical and Animal Care Committee (N°84 COMETHEA, Ethical Committee for Animal Experimentation Poitou-Charentes, France) and by the French ministry (agreement number: 2015072717461531 to Pr Guylène Page as designer of experimental projects on animals for scientific purposes) and agreement APAFIS#13481-2018.013115261004.V3 for imaging experiments. At weaning, all mice were genotyped by polymerase chain reaction (PCR) analysis of tail biopsies according to the manufacturer’s recommended protocols. All animal care and experimental procedures conformed with the French Decree number 2013–118, 1 February 2013 NOR: AGRG1231951D in accordance with European Community guidelines (directive 2010/63/UE). All efforts were made to minimize animal suffering, as well as the number of animals used. The animals were housed in a conventional state under adequate temperature (23 ± 3°C) and relative humidity (55 ± 5%) control with a 12/12 h reversed light/dark cycle with access to food and water *ad libitum*. Throughout the study, the general state of health of the mice was evaluated weekly by monitoring their body weight, food, and water intake.

For this study 27 AD mice (13 males and 14 females) and 24 WT mice (12 males and 12 females) were used. The mice were treated from 7 to 11 months of age by a weekly intraperitoneal i.p. injection of either *trans* ε-viniferin (Vini) at 20 mg/kg (4 AD males, 5 AD females, 4 WT males, and 4 WT females), *trans-*resveratrol (Resv) at 20 mg/kg (4 AD males, 5 AD females, 4 WT males, and 4 WT females) or their vehicle, the polyethylene glycol 200 (PEG 200) at 1.67 mL/kg (5 AD males, 4 AD females, 4 WT males, and 4 WT females). This dose of resveratrol and viniferin was chosen by taken into account the results published in a first study (Caillaud et al., 2019). All treatments were diluted in 0.9% NaCl. At the beginning of the study and at the end of each treatment, behavioral test was performed. PET-scan imaging was only performed at the end of each treatment. Finally, 1 week after PET-scan imaging, the mice were euthanized. The mice were transcardially perfused with 4% paraformaldehyde (PFA) after deep anesthesia with a mix of ketamine (100 mg/kg) and xylazine (10 mg/kg). Brains were rapidly removed, and the right hemisphere was immediately placed in 4% PFA at 4°C for immunofluorescence imaging. In the left hemisphere, the hippocampus was dissected and homogenized for biochemical experiments. All samples were stored at −80°C until further experiments. In all groups (PEG, Resv, Vini), some mice prematurely died. Moreover, the volume of hippocampal lysates was low and did not enable all the signals to be detected by western blot for some mice. Thus, the number of animals was variable for different experimental procedures as indicated in the figure captions.

At the same time, another set of experiments included mice treated weekly with either Vini, Resv, or PEG at the same i.p. doses indicated above from 3 to 12 months of age. Micro-PET imaging was not carried out for financial reasons. Thus, the results concerning amyloid deposits by immunofluorescence and inflammatory parameters [GFAP and IBA1 by western blot and IL-1β by enzyme-linked immunosorbent assay (ELISA)] were in Supplementary Material as indicated in the section “Results.”

### Morris Water Maze

For the behavioral test by the Morris water maze (MWM), the mice were individually placed in a round pool (1 m diameter, 0.5 m height) filled with crushed white chalk mixed with water at room temperature (RT). An escape platform (5 cm diameter) was placed at 15 cm from the edge of the tank in the northeast quadrant of the pool. This platform was submerged under the water surface and was not visible by the mice. During the entire test, this pool was placed in a quiet room with visual cues. A camera recorded the mice’s pathway and behavior. The test consisted of four consecutive daily training periods followed by the retention test on the 5th day. For the training period, before the first trial, the mice were placed on the submerged platform for 5 s. They were tested four trials *per* day, with different starting locations, following a defined sequence. Each trial had a maximum duration of 60 s. If the mouse did not find the escape platform in 60 s, it was physically placed on the platform to learn for 20 s. For the retention test, on the 5th day, the platform was removed, and the mice were allowed to swim for 60 s. The tendency of the mice to search for the platform was evaluated by quantifying the time spent in the target quadrant where the escape platform had previously been located. The time spent by each mouse in the target quadrant and the distance traveled by the mice were recorded and processed using a video tracking device and computer-equipped analytics management system (Kinovea.org software, 0.8.15). The MWM protocol was applied twice, the first time before the first injection, at 7 months of age, and the second time after the last injection, at 11 months. To evaluate the effects of the molecules on the cognitive status of the mice, we assessed the evolution of their performance in the retention test between 7 (before initiation of the treatment) and 11 months (at the end of the treatment). Thus, for each mouse, this evolution was quantified by the following formula:


(1) $$\frac{\begin{array}{c} \mathrm{time}\,\mathrm{spent}\,\mathrm{in}\,\mathrm{the}\,\mathrm{target}\,\mathrm{quadrant}\,\left(11\,\mathrm{months}\right)-\mathrm{time}\,\mathrm{spent}\,\mathrm{in}\,\mathrm{the}\,\mathrm{target}\,\mathrm{quadrant}\,\left(7\,\mathrm{months}\right) \end{array}}{\mathrm{time}\,\mathrm{spent}\,\mathrm{in}\,\mathrm{the}\,\mathrm{target}\,\mathrm{quadrant}\,\left(7\,\mathrm{months}\right)}$$

The motor capacities of the mice were evaluated by quantifying the average swimming speed at 7 and 11 months.

### Positon Emission Tomography-Scan Imaging

The β-amyloid load and TSPO imaging were assessed using [^18^F]Florbetaben and [^18^F]DPA-714, respectively, which were prepared as described previously (Wang et al., 2011) and obtained with a mean molar activity of 50–150 GBq/μmol. Each animal was explored with both tracers with a minimum delay of 3 days between each imaging experiment, according to a paper previously published (Sérrière et al., 2015). The mice received an i.v. injection of either [^18^F]Florbetaben or [^18^F]DPA-714 (15–20 MBq) in the tail vein under gas anesthesia (1.5–2% isoflurane in 1.5–2 L/min of O_2_). A 5 min CT-scan was first acquired for 5 min for attenuation correction, and the animals were then scanned over 61 min using a Super Argus PET/CT system (Sedecal, Spain). PET images were rebinned into 33 frames, and the last 30 min acquisition was extracted for reconstruction, and then analysis using PMOD (3.403, PMOD Technologies, Zurich, Switzerland)^1^. Then, a voxel-based analysis was used to assess the differences in radiotracer binding between the averaged brains of Vini vs. Resv vs. PEG treated mice. This was performed using unpaired Student’s two-tailed *t*-tests with *p*-values corrected for multiple comparisons using the Benjamini-Hochberg control of false discovery rate (Benjamini and Hochberg, 1995). However, all the individual voxel comparisons missed significance, as described in other PET studies with low degrees of freedom (Endepols et al., 2010). Therefore, Z-score maps with a threshold of *p* = 0.05 for uncorrected *p*-values were generated. The regions of interest were derived from Mirrione’s templates using PMOD v3.2 software and applied to Z-score maps to obtain the Z-score values in the regions of interest (ROI).

### Immunofluorescence

After 24 h in 4% paraformaldehyde at 4°C, right brain hemispheres were rinsed in PBS (phosphate buffer saline), dehydrated, and embedded in paraffin for sagittal sectioning (4 μm thickness). Sagittal sections were cut in a microtome (Microm Microtech, Brignais, France) and mounted on Super-Frost Plus 1 slides (CML, Nemours, France) with water and conserved at 4°C until use. Immunolabelings were performed as previously described (Damjanac et al., 2007; Couturier et al., 2012), using specific antibodies at the dilutions indicated in Supplementary Table 2. Multiple labeled samples (two slices *per* mice) were examined with an Olympus BX51 epifluorescent optical microscope. Images were blind analyzed with ImageJ, as previously described (Caillaud et al., 2019). Multiple labeled samples were also examined with a spectral confocal FV-1000 station installed on an inverted microscope IX-81 (Olympus, Tokyo, Japan) with an Olympus x60 oil, 1.2 NA, objective lens with an optical section separation (z-interval) of 0.3 μm. Fluorescence signal collection, image construction, and scaling were performed using the control software (FluoView FV-1000, 4.2.1.20 Olympus). Multiple fluorescence signals were acquired sequentially to avoid crosstalk between image channels. Fluorophores were excited with the 405 nm line of a diode (for DAPI), and the 543 nm line of a HeNe laser (for TRITC). Emitted fluorescence was detected through spectral detection channels and images were merged as an RGB image.

### Biochemical Experiments

#### Preparation of Protein Lysates

After euthanasia, the hippocampus of the left hemisphere was dissected and homogenized for biochemical experiments as previously described (François et al., 2014) in 10 volumes of lysis buffer (25 mM Tris–HCl, 150 mM NaCl, 1 mM EDTA, pH 7.4) and supplemented with 50 mM NaF, 1 mM PMSF, protease and phosphatase inhibitor cocktails (50 μL/g of tissue and 10 μL/mL of lysis buffer, respectively). Before use, supernatants were stored at −80°C. For ELISA experiments, requiring the use of an insoluble fraction, the pellet was suspended with 30 μL of lysis buffer before treatment with guanidine as explained below.

#### Western Blots

For each sample, 20 μg proteins were denatured in Laemmli Sample Buffer containing DTT by boiling during 5 min. Electrophoreses in 4–20% Tris-Glycine gels were performed according to the manufacturer’s recommendations (at 150 V during 40 min in Tris-Glycine SDS Running Buffer). Proteins were then transferred to nitrocellulose membranes using the Trans-Blot Turbo system set to program 25 V for 7 min. The membranes were washed for 10 min in Tris-buffered saline/Tween (TBST: 20 mM Tris–HCl, 150 mM NaCl, pH 7.5, 0.05% Tween 20) and non-specific antigenic sites were blocked 2 h in TBST containing 5% semi-skimmed milk and 0.21% sodium fluoride. The membranes were incubated with primary antibodies at the adequate dilution (Supplementary Table 2) overnight at 4°C. Then, they were washed twice with TBST and incubated with the HRP-conjugated secondary antibodies (Supplementary Table 2) during 1 h at RT. The membranes were washed again and exposed to the chemiluminescence Luminata Forte Substrate. After two washes in TBST, the membranes were put in contact with mouse antibodies against β-actin overnight at 4°C, washed with TBST and incubated with HRP-conjugated secondary antibodies for 1 h before being exposed to the chemiluminescence Luminata Classico Substrate. All luminescent signals were captured by the Gbox system (GeneSnap software, Syngene, Ozyme distributor). Automatic image analysis software was supplied with Gene Tools (Syngene, Ozyme distributor). In each case, the protein of interest/β-actin *ratios* were calculated. Results were then normalized as a percentage of expression in PEG treatment for both groups (WT and AD).

#### Aβ40-42 ELISA

The levels of Aβ_42_ and Aβ_40_ were quantified using an ELISA kit (Gibco-Invitrogen). Concerning the insoluble fraction, the pellets obtained after the preparation of hippocampal homogenates from the left hemisphere were suspended with 30 μL of lysis buffer. Then, a homogenization with 8 volumes of guanidine–Tris buffer (5 M guanidine HCl/50 mM Tris–HCl, pH 8.0) was performed in order to extract insoluble Aβ. Homogenates were incubated at RT for 4 h before they were assayed. Samples were diluted in cold BSAT-DPBS reaction buffer (0.2 g/L KCl, 0.2 g/L KH_2_PO_4_, 8.0 g/L NaCl, 1.15 g/L Na_2_HPO_4_, 5% BSA, 0.03% Tween-20, pH 7.4) supplemented with Protease Inhibitor Cocktail. Samples were centrifuged at 16,000 *g* for 20 min at 4°C. The supernatant was diluted in the standard diluent buffer available in the kit. The final concentration of AEBSF (included in the protease inhibitor cocktail) was 1 mM in order to prevent proteolysis of Aβ peptides. The human Aβ_42_ and Aβ_40_ standards were also diluted in the same standard diluent buffer as the samples. The plates were incubated with detection antibody overnight at 4°C. Concerning the soluble fraction, the same homogenization steps with guanidine and dilutions were performed. After washing, the plates were incubated with HRP anti-rabbit antibodies for 30 min at RT, then they were washed, and stabilized chromogen was added in each well for 30 min in a dark chamber at RT. After stopping the reaction, the absorbance of the plates was read at 450 nm using the Multiskan spectrum spectrophotometer. The standard curves were established using a range of concentrations (15.63–1,000 pg/mL) of a synthetic Aβ_42_ peptide, and a range of concentrations (7.81–500 pg/mL) of a synthetic Aβ_40_ peptide. Data are expressed as pg of total Aβ_40–42_/mg of protein.

#### IL-1β ELISA

Homogenates from mouse hippocampus were analyzed for IL-1β levels using ELISA kit (sensitivity: 16 pg/mL) according to the manufacturer’s instructions (BioLegend, Ozyme, Saint-Quentin-en-Yvelines, France). The range of IL-1β analysis was between 31.3 and 2,000 pg/mL. The cytokine levels were calculated by plotting the optical density (OD) of each sample against the standard curve. The intra- and inter-assay reproducibility was >90%. OD values obtained for duplicates that differed from the mean by greater than 10% were not considered for further analysis. For convenience, all results are expressed in pg/mg protein.

### Statistical Analysis

Results were expressed as means ± standard error (SEM). The statistical program GraphPad (GraphPad Software, San Diego, CA, United States) was used to compare quantitative variables. For all comparisons, the level of significance was *p* < 0.05. To compare quantitative variables between two groups of mice, Mann–Whitney test was used. Comparisons between all groups of mice were performed using a Kruskal–Wallis test followed by a *post hoc* Dunn’s test. For imaging experiments, inter-group comparison was performed using a two-tailed unpaired Student *t*-test. Effect size was evaluated for each significant difference observed and expressed as *d*-values corresponding to a large effect size for *d* values superior to 0.80 (Cohen, 1988). The signals extracted using the ROIs on the z-score maps were considered for further analysis when representing at least 50 contiguous voxels for a statistical threshold set at *p* < 0.05.

## Results

### Reduction of the Hippocampal Amyloid Burden and Deposits by Viniferin

**FIGURE 1:**
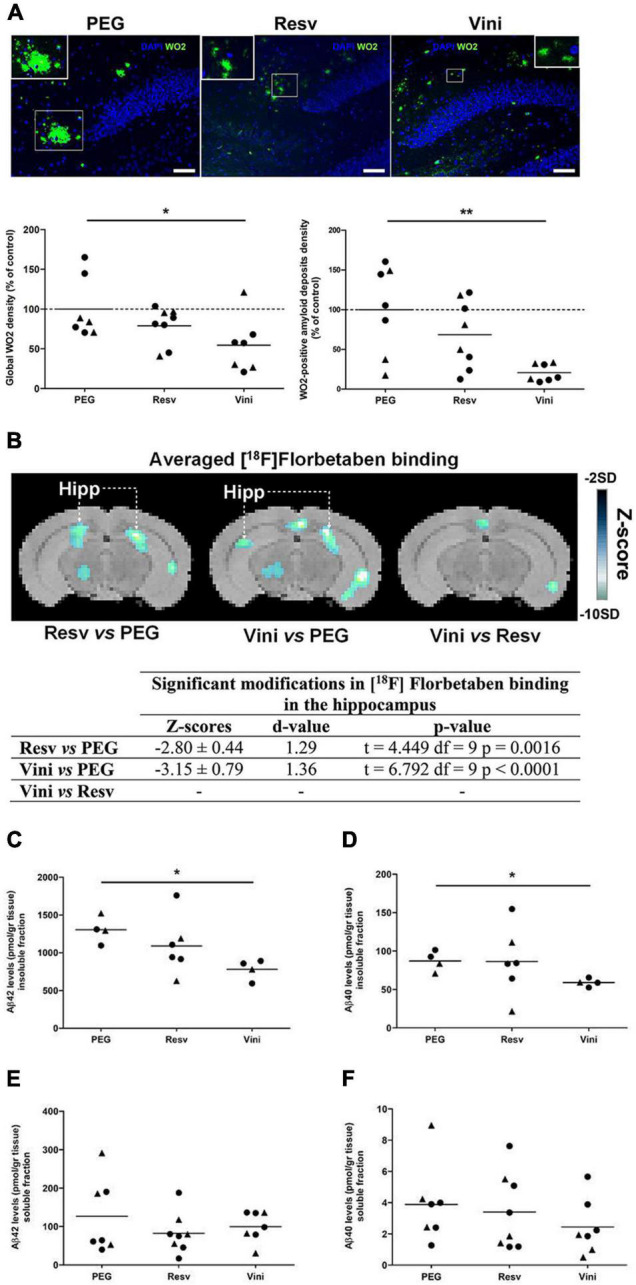
Effects of resveratrol and viniferin on the hippocampal amyloid deposits. The double transgenic APPswePS1dE9 mice were intraperitoneally treated with resveratrol, *trans* ε-viniferin or their vehicle (PEG 200) at 20 mg/kg/week from 7 to 11 months of age. **(A)** The senile plaques were stained with antibody against amyloid peptide (clone WO2, green) and the nuclei with DAPI (blue). On each image, a magnification delineated by a white frame was added. Scale bars: 50 μm. The quantification of global signal WO2 and of WO2-positive amyloid deposits were represented. **(B)** Coronal images of significant differences in [^18^F]Florbetaben binding (z-score maps; decreases in tracer binding from dark to white; Student unpaired *t*-test; *p* < 0.01) in AD mice fused with an MRI template in three conditions: from left to right Resv vs. PEG, Vini vs. PEG, and Vini vs. Resv. Table resuming the statistical differences in [^18^F]Florbetaben binding for the three treatment conditions. The z-score and *d*-value are presented for each significant difference observed along with the *t*, df, and *p* values of the unpaired *t*-test. Hipp, hippocampus; PEG, polyethylene glycol 200; Resv, *trans*-resveratrol; Vini, *trans* ε-viniferin. **(C–F)** The hippocampal levels of insoluble fraction **(C)** and soluble fraction **(E)** of Aβ42 and those of Aβ40 **(D,F)** were quantified using ELISA. The dotted line represents the mean of the PEG group or 100% of PEG signal. The results were expressed as percentage of control (rounds represent females, triangles represent males). To compare values between PEG- and polyphenol-treated-mice (by *trans* resveratrol or *trans* ε-viniferin), Kruskal–Wallis test followed by Dunn’s test or the Mann–Whitney test for two groups were used, (*n* = 4–8). **p < 0.01, *p < 0.05 compared to PEG-treated AD mice (control mice).

In this study in which animals were treated from 7 to 11 months of age, amyloid deposits in the hippocampus were visualized by immunofluorescence, using the monoclonal mouse anti-amyloid WO2 clone, which recognizes amino acid residues 4–10 of human Aβ. The hippocampal amyloid plaques in viniferin-treated AD mice appeared to be less compact and intense than those of PEG-treated mice (Figure 1A). The global amyloid burden quantified by immunofluorescence in the hippocampus of AD mice showed a significant 45.5% decrease with viniferin compared to PEG-treated mice (Figure 1A). When the image analysis focused only on aggregated amyloid plaques, viniferin significantly reduced (−79.5%) the density of hippocampal amyloid deposits in these AD mice (Figure 1A). Resveratrol did not induce a significant decrease in either the parameters in the hippocampus compared to PEG-treated AD mice (Figure 1A). Interestingly, mice that they had received an earlier treatment of viniferin from 3 months and over a longer period (9 months) showed a significant decrease in global amyloid burden and WO2-positive amyloid deposits, compared to PEG-treated mice (−35 and −41.5%, respectively). Furthermore, viniferin had a better activity compared to resveratrol (decrease of 31 and 36%, respectively), (Supplementary Figure 1).

The PET-Scan analysis of [^18^F]Florbetaben binding in the hippocampus showed a significant decrease both in viniferin- (*p* < 0.0001) and resveratrol-treated mice (*p* = 0.0016) compared to controls (Figure 1B).

These results were completed by quantification of insoluble and soluble Aβ40 and Aβ42 levels in the hippocampus. Compared to the PEG-treated AD mice, the levels of both Aβ42 and Aβ40 insoluble fractions were significantly reduced by 40 and 32%, respectively (Figures 1C,D) in the hippocampus of viniferin-treated mice while no significant differences were observed in resveratrol-treated mice, or between resveratrol- and viniferin-treated mice. No significant difference in soluble Aβ42 and Aβ40 was observed between all groups (Figures 1E,F).

Furthermore, the global WO2 density significantly decreased in cortex of viniferin-treated mice compared to resveratrol-treated and control mice (−78.6 and −74.5%, respectively) (Supplementary Figure 2).

### Effect of Viniferin on the Reactivity of Microglia

**FIGURE 2:**
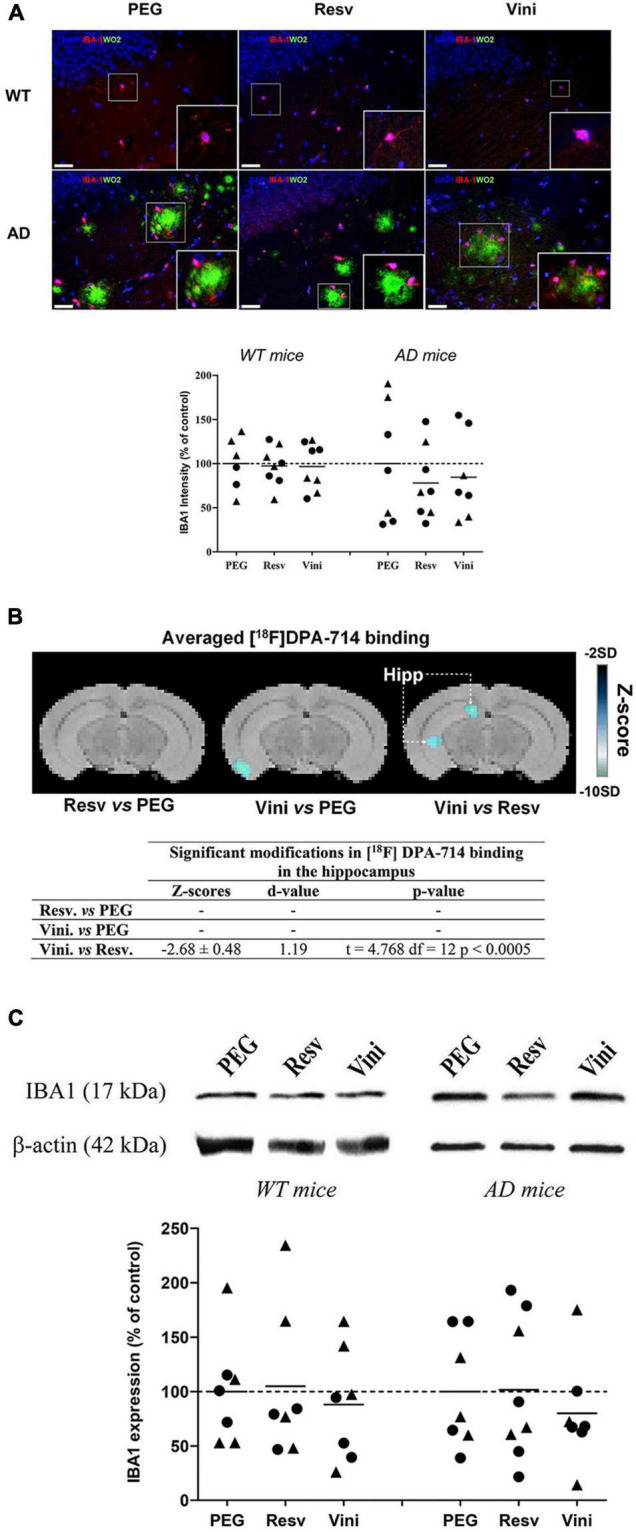
Effects of resveratrol and viniferin on the microglia reactivity. The WT mice and the double transgenic APPswePS1dE9 mice were treated by resveratrol, *trans* ε-viniferin or their vehicle (PEG 200) intraperitoneally from 7 to 11 months of age. **(A)** The senile plaques were stained with antibody against amyloid peptide (clone WO2, green), microglia with antibody against IBA1 (red), and the nuclei with DAPI (blue). On each image, a magnification delineated by a white frame was added. The immunofluorescent IBA1 signal was quantified as described in section “Materials and Methods.” Scale bars: 25 μm. **(B)** Coronal images of significant differences in [^18^F]DPA-714 binding (z-score maps; decreases in tracer binding from dark to white; Student unpaired *t*-test; *p* < 0.01) in AD mice fused with an MRI template in three conditions: from left to right Resv vs. PEG, Vini vs. PEG, and Vini vs. Resv. Table resuming the statistical differences in [^18^F]DPA-714 binding for the three treatment conditions. The z-score and *d*-value are presented for each significant difference observed along with the t, df and *p* values of the unpaired *t*-test. Hipp, hippocampus; PEG, polyethylene glycol 200; Resv, *trans*-resveratrol; Vini, *trans* ε-viniferin. **(C)** Cropped blots of IBA1 and corresponding β-actin were shown and IBA1/β-actin *ratio*s were calculated. The means of IBA1 levels were represented for all groups. The results were expressed as percentage of PEG-treated mice as controls (rounds represent females, triangles represent males). The dotted line represents 100% signal. To compare values between PEG- and polyphenol-treated-mice (by *trans* resveratrol or *trans* ε-viniferin), Kruskal–Wallis test followed by Dunn’s test were used (*n* = 6–8). Any significant result was observed in **(A,C)**. Full-length blots are presented in Supplementary Figure 4.

The reactivity of hippocampal microglial cells was assessed by quantifying the immunolabeling of IBA1 (Figure 2A). The intensity of this marker was unchanged in all groups of WT mice (Figure 2A). In AD mice, however, both resveratrol and viniferin induced a low reduction in the IBA1 signal compared to the PEG group (−22 and −15.5%, not significant, respectively). However, the PET-Scan analysis of the TSPO tracer [^18^F]DPA-714 binding, which is an index of microglia reactivity in the hippocampus, showed a significant decrease between viniferin- and resveratrol-treated mice in the hippocampus (Figure 2B) although no difference in the tracer accumulation was observed between the viniferin or resveratrol and PEG groups (Figure 2B). IBA1 immunoreactivity was similar in all WT and AD groups despite a non-significant decrease of 21.6% in IBA1 expression in viniferin-treated AD mice compared to resveratrol-treated AD mice (Figure 2C). In AD mice treated between 3 and 12 months, no change was observed for IBA1 immunoreactivity (Supplementary Figure 3B).

### Effect of Viniferin on the Reactivity of Astrocytes

**FIGURE 3:**
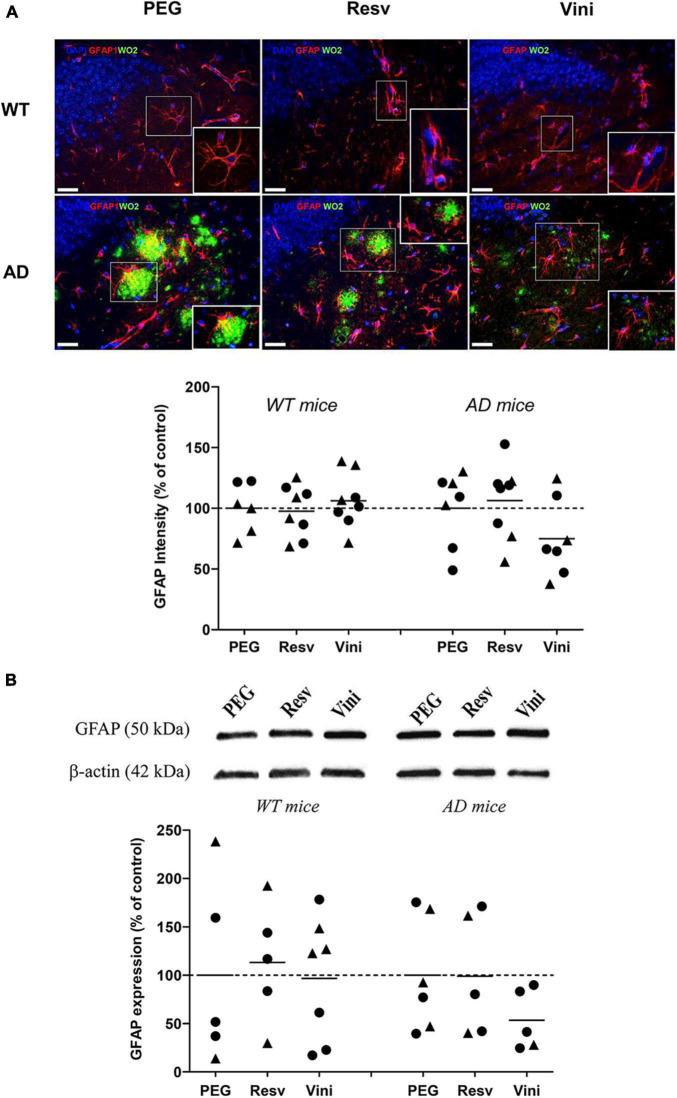
Effects of resveratrol and viniferin on the reactivity of astrocytes. The WT mice and the double transgenic APPswePS1dE9 mice were treated with resveratrol, *trans* ε-viniferin or their vehicle (PEG 200) intraperitoneally from 7 to 11 months of age. **(A)** The senile plaques were stained with antibody against amyloid peptide (clone WO2, green), astrocytes with antibody against GFAP (red), and the nuclei with DAPI (blue). On each image, a magnification delineated by a white frame was added. The immunofluorescent GFAP signal was quantified as described in section “Materials and Methods.” Scale bars: 25 μm. **(B)** Cropped blots of GFAP and corresponding β-actin were shown and GFAP/β-actin *ratios* were calculated. The means of GFAP levels were represented for all groups. The results were expressed as percentage of PEG-treated mice as controls (rounds represent females, triangles represent males). The dotted line represents 100% signal. To compare values between PEG- and polyphenol-treated-mice (by *trans* resveratrol or *trans* ε-viniferin), Kruskal–Wallis test followed by Dunn’s test were used (*n* = 4–8). Any significant result was observed. Full-length blots are presented in Supplementary Figure 4.

The reactivity of hippocampal astrocytes was assessed by quantifying the immunolabeling of GFAP (Figure 3A). As observed in microglia, the GFAP signal did not differ among the different groups, even if a decrease was found in the AD viniferin-treated group vs. the AD PEG-treated and AD resveratrol-treated groups (−25%, *p* = 0.156 and −30%, *p* = 0.073, respectively). GFAP immunoreactivity decreased in viniferin-treated mice compared to vehicle- or resveratrol-treated mice (−46.5 and −46%, not significant, respectively), whereas it was similar in all WT groups (Figure 3B). In AD mice treated between 3 and 12 months, the GFAP immunoreactivity decreased in AD mice treated with viniferin although this was not statistically significant (−27% compared to PEG-treated mice) (Supplementary Figure 3C).

### Effect of Viniferin on IL-1β Production

**FIGURE 4:**
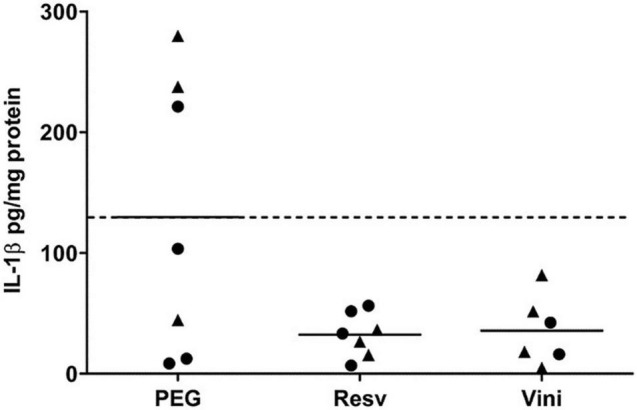
IL-1β levels in hippocampus after the resveratrol or viniferin treatment. As indicated in section “Materials and Methods,” IL-1β was quantified by ELISA in hippocampal homogenates of AD mouse brain. Means were represented and expressed in pg/mg protein (*n* = 6–7). Kruskal–Wallis test followed by Dunn’s test were used but any significant result was observed. The dotted line represents the mean of the PEG group. Black rounds represent females and black triangles represent males.

Results showed that both resveratrol and viniferin induced a reduction in IL-1β levels in AD mice treated from 7 to 11 months (−75 and −72.5%, not significant, respectively) (Figure 4). Moreover, this reduction was also observed in the hippocampus of AD mice treated with resveratrol and viniferin from 3 to 12 months (−42 and −37%, not significant, respectively) (Supplementary Figure 3A).

### Partial Improvement of Spatial Memory Decline by Polyphenols in Alzheimer’s Disease Mice

**FIGURE 5:**
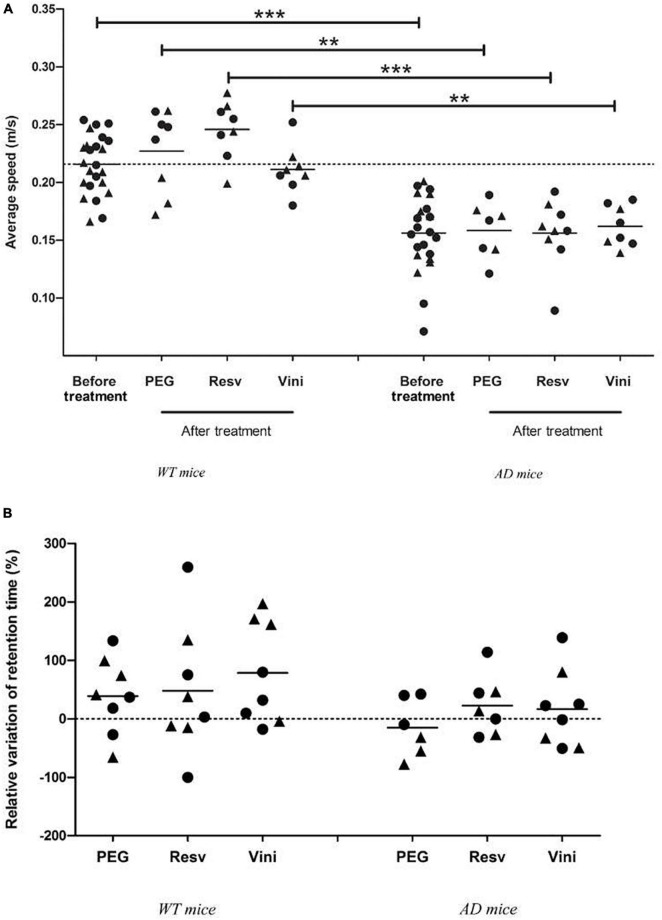
Effects of resveratrol and viniferin on the motor capacities and memory decline. **(A)** The average speed of WT and AD mice was measured before and after treatment. **(B)** The relative variation of retention time expressed as percentage between the beginning and the end of treatment was quantified for WT and AD mice. To compare values of average speed and relative variation of retention time, the Mann–Whitney test was used (*n* = 7–24). ***p < 0.001, **p < 0.01, compared to each treatment between WT and AD treated mice. The dotted line represents the mean of average speed before treatment (panel A) and sets at 0 for relative variation of retention time (panel B). Black rounds represent females and black triangles represent males.

To evaluate the spatial memory of mice, the MWM test was performed, before the beginning of treatment (at 7 months) and after the last injection (at 11 months). For each WT and AD mouse group, no difference in swimming speed was observed between the start and the end of the study (Figure 5A). Neither the treatment nor aging induced a modification in the motor capacities of mice in the two groups. However, one may note that AD mice presented a significantly lower swimming speed than WT mice after the onset of the study (Figure 5A). Finally, after analyzing the relative variation in the retention time between 7 and 11 months, it appeared that WT mice presented a similar positive variation in all three groups. In vehicle-treated AD mice, a negative relative variation (−15%) was observed whereas in resveratrol- and viniferin-treated animals, this variation of the retention time between the start and the end of the treatment became positive (+20%, Figure 5B).

## Discussion

The aim of this work was to compare the effects of resveratrol and viniferin in a murine model of AD displaying some histopathological lesions of this disease. These polyphenols were evaluated on the amyloid deposits, the reactivity of microglia and astrocytes, the IL-1β production and cognitive decline. We already showed in this model that viniferin administered at 10 mg/kg by i.p. injection once a week from 3 until 6 months of age, partially prevented the formation of amyloid deposits, and decreased the reactivity of astrocytes and microglia (Caillaud et al., 2019). However, these results only demonstrated the preventive effects of this stilbene on APPswePS1dE9 mice and were not compared to those of resveratrol. Indeed, these transgenic mice displayed diffuse amyloid deposits from 4 months of age with a robust neuroinflammation and cognitive impairment at 12 months (Savonenko et al., 2005; Garcia-Alloza et al., 2006; Ruan et al., 2009), supporting the preventive effect of viniferin on the appearance of amyloid deposits (Caillaud et al., 2019). Thus, in the present study, AD and WT mice were treated by resveratrol or viniferin, at 20 mg/kg, or their vehicle, PEG-200, by i.p. injections once a week from 7 until 11 months, when amyloid deposits were already present. Moreover, it was necessary to determine the effects of viniferin on memory decline in comparison to resveratrol.

Like many models of mice modeling Alzheimer’s disease, these APPswePS1dE9 mice display amyloid deposits in the whole brain including the cerebellum with age (Sérrière et al., 2015). Here, we compared data obtained in the hippocampus by immunofluorescence assay and micro-PET imaging. The classic AD pattern consists of early prominent involvement of the entorhinal cortex and the hippocampus. Recently, authors found by using different tools that medial temporal lobe atrophy including hippocampus followed by ventricular enlargement are two mid-life physiopathological events characterizing AD brain (Coupé et al., 2019; Chauveau et al., 2021). Thus, we targeted the hippocampus in this study.

In this mouse model of AD, it has already been shown that resveratrol (16 mg/kg/day) reduced thioflavin S-positive senile plaque after 10 months of treatment (Porquet et al., 2014). In the same animal model, immunohistochemistry results showed that viniferin at 20 mg/kg/week during 4 months and 9 months significantly reduced amyloid deposits in the hippocampus while resveratrol did not. Thus, viniferin seems to decrease amyloid deposits more quickly than resveratrol. Amyloid deposits in these mice were also investigated using micro-PET imaging with [^18^F]Florbetaben. Previous studies using this tracer showed an increased radioactivity in Tg vs. WT age-matched living mice from 12 months of age in the cortex (Rominger et al., 2013) and hippocampus (Stenzel et al., 2019). Using a quantification method that we previously developed (Nicolas et al., 2017), the present PET data showed that viniferin but also resveratrol decreased [^18^F]Florbetaben binding in the hippocampus after 4 months of treatment. It has been shown that [^18^F]Florbetaben is a reliable index of β-amyloid load as it has been shown to have a better affinity for dense fibrillary than for diffuse plaques (Catafau et al., 2016). Thus, the apparent discrepancy between immunofluorescence and *in vivo* PET experiments could be related to the fact that the PET-scan analysis considers the entire volume of brain regions, whereas only brain sections were analyzed by immunofluorescence.

We also showed that viniferin treatment decreased insoluble forms of Aβ, whereas resveratrol treatment did not show significant results. The insoluble forms are generated from the β-sheet and give the amyloid protein its compact, stable, and insoluble appearance (Shankar et al., 2008; Hong et al., 2014; Morris et al., 2014). Therefore, the significant decrease in the insoluble fraction in viniferin treated mice agrees with the decrease in amyloid deposits observed by immunofluorescence and by PET-scan imaging. This result was previously described *in vitro*, showing that viniferin induced the disaggregation the Aβ42 peptide (Vion et al., 2018). Resveratrol did not show a significant effect on the insoluble fraction which again agrees with the previous results. However, *in vitro*, resveratrol is known to not prevent oligomer formation (Feng et al., 2009). These oligomers are an association of peptides which can adopt heterogeneous morphologies, complicating their analysis. Thus, we find in the literature under the name of oligomers not only small oligomers (dimer/trimer/tetramer/pentamer/hexamer), larger oligomers (dodecamer: Aβ56) but also protofibrils and fibrils of which the number of monomers constituting them is unknown (Huang et al., 2000; Walsh and Selkoe, 2007; Amar et al., 2017). In the progression of AD, soluble monomeric Aβ peptides are recognized as the primary toxic forms. It has been proved for a long time that the soluble Aβ oligomers (AβOs), isolated from AD patients’ brains, reduced the number of synapses, inhibited long-term potentiation, and enhanced long-term synaptic depression in brain regions of memory in animal models of AD (Lambert et al., 1998; Klein et al., 2001; Lacor et al., 2007). Several studies hypothesized that the soluble AβOs could be sequestered within Aβ plaques until they reach a maximum saturation. Then, AβOs diffuse onto synaptic membranes, leading to a harmful cascade that damages neurons and synapses. Although this decrease was not found in the soluble fraction, it is important to emphasize that both soluble Aβ40 and Aβ42 levels did not increase with the treatments and that viniferin was able to reduce insoluble forms of Aβ more effectively than resveratrol. These results can be partially supported by the physical interaction of viniferin with amyloid peptide, since it has been proved that viniferin interferes with the β-sheet to exert its activity (Francioso et al., 2015; Freyssin et al., 2018).

Increasing evidence suggests that neuroinflammation contributes to the pathogenesis of AD. In APPswePS1dE9 mice, clusters of astrocytes and microglia were observed in the hippocampus and cortex from 6 months of age, and continued to progress with age (Ruan et al., 2009). Moreover, the activation and accumulation of glial cells and astrocytes around amyloid plaques has already been studied and described in AD patients (Overmyer et al., 1999; Prokop et al., 2013; Heppner et al., 2015). To assess the action of viniferin in AD on glial cells, the use of IBA1 and TSPO markers expressed in reactive microglia, and GFAP and TSPO markers expressed in reactive astrocytes were used. Furthermore, IL-1β was quantified in hippocampal homogenates. It is known that IL-1β is synthesized and released by both activated microglia and astrocytes in a pro-form, i.e., pro-IL-1β which is cleaved by caspase-1 in the cytoplasm in response to a variety of stimuli. It is a member of the IL-1 cytokine family and is considered as a major proinflammatory cytokine in the brain and plays a key role in the progression of AD (Wang et al., 2015; Ising et al., 2019; Webers et al., 2020). In the present study, no significant difference was observed for GFAP and IBA1 markers in WT mice whatever the treatment although a non-significant reduction was observed in viniferin-treated AD mice. These results disagree with the literature, where GFAP and IBA1 levels are reported to be significantly reduced by resveratrol (Bastianetto et al., 2015; Cheng et al., 2015; Yao et al., 2015). The evaluation of TSPO density is now well-recognized as a relevant index of microglial activation (Chen and Guilarte, 2008) and a great number of literature data have to date reported an upregulation of this biomarker in Alzheimer’s disease (Tournier et al., 2020). We measured TSPO density by PET imaging using [^18^F]DPA-714 in each mouse previously explored with [^18^F]Florbetaben, as already performed in this animal model (Sérrière et al., 2015). We did not detect any modification of the signal in the two groups of treated vs. control mice, but interestingly, a significant decrease in this signal was measured in the viniferin-treated group vs. the resveratrol-treated group. While TSPO PET imaging has already made it possible to longitudinally follow the neuroinflammatory process in Tg mice models of AD (Sérrière et al., 2015; Takkinen et al., 2017; Chaney et al., 2018), the present study demonstrates for the first time that such an *in vivo* method is suitable for evaluating long-term treatment effects. Further experiments would be needed to clarify the cellular origin of this over-expressed protein that is known to originally come mainly but not exclusively from activated microglia (Nutma et al., 2021). The absence of a significant decrease in inflammatory parameters (GFAP, IBA1, and IL-1β) by immunofluorescence and western blot could be related to a recently discovered side effect of the vehicle, PEG 200. We recently demonstrated that PEG 200, administered i.p. once a week at the dose of 1.67 mL/kg, induced hippocampal inflammation in WT mice (Freyssin et al., 2021), showing that the chronical administration of this molecule led to an increase of both GFAP, IBA1, and IL-1β expressions in the hippocampus of WT mice (Freyssin et al., 2021). However, the PET imaging exploration demonstrated a significant decrease in [^18^F]DPA-714 tracer binding by viniferin compared to resveratrol. In this study, the use of IBA1 as the main marker of microglia is questionable. IBA1 stains both resting and activated cells (Walker and Lue, 2015). Several studies used CD68, a common marker for macrophage lineage cells and primarily localized in microglia, and perivascular macrophages (Hopperton et al., 2018). CD68 labels the lysosome and is therefore commonly considered as a marker of activated phagocytic microglia. Its expression was positively correlated to cognitive decline (Minett et al., 2016). The use of this marker would be more judicious to compare these results with the TSPO used in PET-scan imaging, as previously done in another study (Sérrière et al., 2015).

During the progression of AD, deficits have been reported across cognitive domains and more precisely in spatial learning (Lalonde et al., 2005). Until 7 months, the spatial memory of AD mice is comparable to that of WT mice, but it is impaired at 12 months as measured by performance in the MWM (Volianskis et al., 2010). In this study, the spatial memory test was conducted to assess learning and spatial memory in AD and WT treated mice. Before treatment and after treatment, a significant difference was observed in the swimming speed between AD and WT mice. These motor impairments in AD mice could coincide with the development of AD related lesions within the motor regions of the CNS, although another study showed no difference in motor function of APPswePS1dE9 mice (Lalonde et al., 2005). In our study, no significant difference was observed in the swimming speed, between 7 and 11 months, for both WT and AD mice, indicating that neither age nor treatment impaired the motor capacities. Moreover, after treatment, the difference in swimming speed between AD and the respective WT groups remained significant, indicating that treatment did not improve swimming speed. Concerning the relative variation in the retention time between 7 and 11 months, a positive value was calculated for WT mice whatever the treatment, suggesting good mnesic learning. On the contrary, a negative variation of this retention time was calculated in the vehicle-treated AD mice, which spent less time in the target quadrant than the polyphenol-treated AD mice. This negative variation could be the sign of cognitive decline found in AD, while treatment by resveratrol or viniferin could partially improve the cognitive decline as described for resveratrol in the literature (Porquet et al., 2013; Chen et al., 2019). However, the absence of significance can be explained by the small number of animals in each group and the variability of the data.

To conclude, viniferin is a polyphenol that possesses greater beneficial properties for AD than resveratrol. A significant decrease in amyloid deposits (−79%) and a partial protection of cognitive decline (+20%) were observed for the first time in an AD mouse model after a chronic treatment. The major target of viniferin is the amyloid deposits with here the demonstration that it has an effect also with its curative treatment on this model of transgenic AD mice. The impact of polyphenols on glial reactivity must be interpreted with caution because PEG 200 was found to induce hippocampal inflammation (Freyssin et al., 2021). However, the significant decrease in the TSPO density measured by PET imaging by viniferin compared to resveratrol once again highlighted that viniferin is also more effective than resveratrol against glial reactivity. In the perspective of a clinical trial, further studies using oral administration of viniferin in a suitable excipient vehicle would be needed to assess its beneficial effects on inflammation.

## Data Availability Statement

The original contributions presented in the study are included in the article/Supplementary Material, further inquiries can be directed to the corresponding author.

## Ethics Statement

The animal study was reviewed and approved by the N°84 COMETHEA, Ethical Committee for Animal Experimentation Poitou-Charentes, France.

## Author Contributions

AF: methodology, investigation, formal analysis, writing – original draft, and review. AR: conceptualization, funding acquisition, co-supervision, and review. BF: supervision and writing – review and editing. LG: validation of data imaging, formal analysis, and review. SS: methodology of micro-PET imaging. CT: formal analysis of data imaging. FP: resources and investigation for extraction and purification of polyphenols. JG: resources, investigation for extraction and purification of polyphenols, and review. SC: funding acquisition, validation of data imaging, and writing – review and editing. GP: conceptualization, co-supervision, resources, methodology, investigation, validation, and writing – review and editing. All authors contributed to the article and approved the submitted version.

## Conflict of Interest

The authors declare that the research was conducted in the absence of any commercial or financial relationships that could be construed as a potential conflict of interest.

## Publisher’s Note

All claims expressed in this article are solely those of the authors and do not necessarily represent those of their affiliated organizations, or those of the publisher, the editors and the reviewers. Any product that may be evaluated in this article, or claim that may be made by its manufacturer, is not guaranteed or endorsed by the publisher.

## Footnotes

1
www.pmod.com
