# Supplementary material for: Trans ε-Viniferin Decreases Amyloid Deposits With Greater Efficiency Than Resveratrol in an Alzheimer’s Mouse Model
Source: Front Neurosci. 2022 Jan 6;15:803927. doi: 10.3389/fnins.2021.803927 (PMC8770934; doi:10.3389/fnins.2021.803927)
Supplement: Supplementary file 2 [file Image_2.pdf]

Supplementary Figure 2

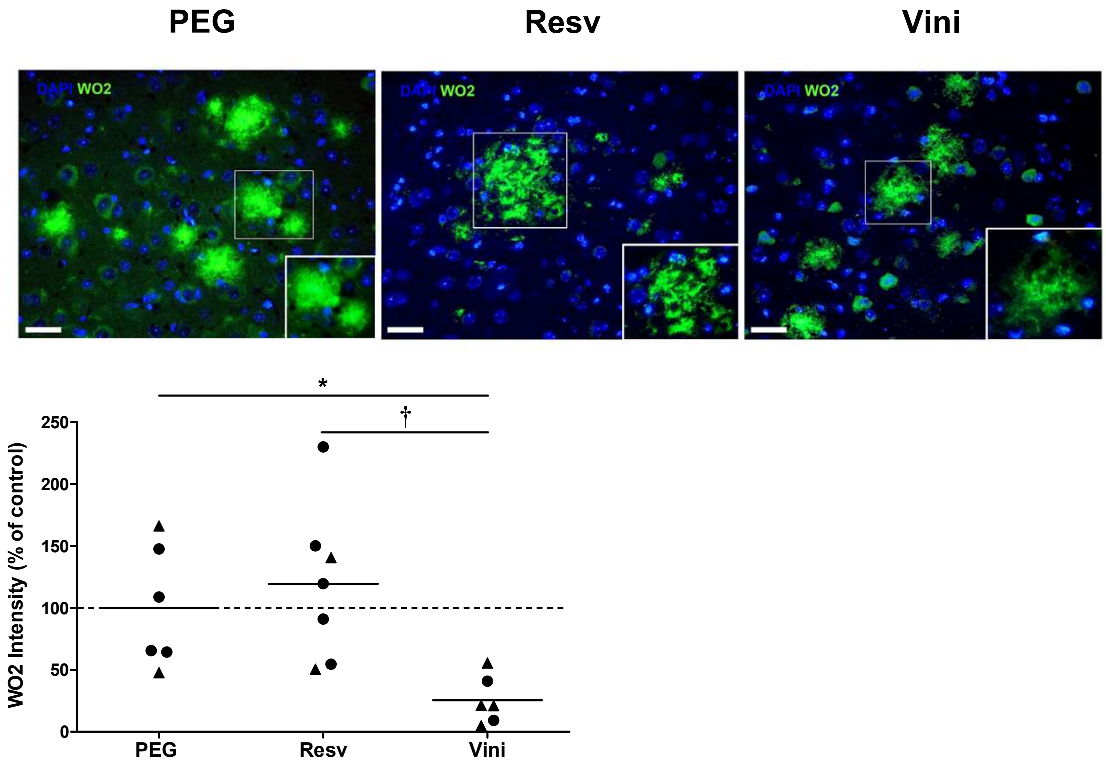

Effects of resveratrol and viniferin on the cortical amyloid deposits after 4 months of treatment. The double transgenic APPswePS1dE9 mice were intraperitoneally treated with resveratrol, *trans* ε-viniferin or their vehicle (PEG 200 as control) at 20mg/kg from 7 to 11 months of age. The senile plaques were stained with antibody against amyloid peptide (clone WO2, green) and the nuclei with DAPI (blue). On each image, a magnification delineated by a white frame was added. Scale bars: 25 μm. The quantification of global signal WO2 was represented. In the graph, the dotted line represents 100% and the results were expressed as percentage of control (rounds represent females and triangles represent males). To compare values between PEG- and polyphenol-treated-mice (by *trans* resveratrol or *trans* ε-viniferin), a Kruskal-Wallis test followed by Dunn's Multiple Comparison Test were used, ($n = 6$ or 7 in each group). $^*p < 0.05$ compared to PEG-treated AD mice (control mice) and $^\dagger p<0.05$ compared to resveratrol-treated mice.
