# Supplementary material for: Trans ε-Viniferin Decreases Amyloid Deposits With Greater Efficiency Than Resveratrol in an Alzheimer’s Mouse Model
Source: Front Neurosci. 2022 Jan 6;15:803927. doi: 10.3389/fnins.2021.803927 (PMC8770934; doi:10.3389/fnins.2021.803927)
Supplement: Supplementary file 3 [file Image_3.pdf]

Supplementary Figure 3

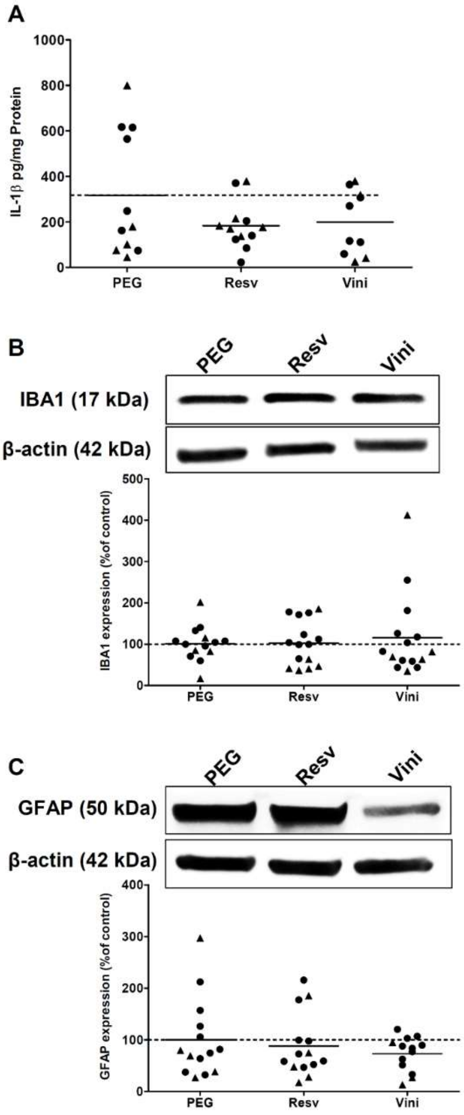

Inflammatory parameters in APPswePS1dE9 mice after 9 months of treatment with resveratrol or viniferin. Panel A: As indicated in material and methods, IL-1β was quantified by ELISA in hippocampal homogenates of AD mouse brain. Means were represented and expressed in pg/mg protein (n = 11, 12 and 10 for PEG, Resv and Vini, respectively). **Panel B:** Cropped blots of IBA1 and corresponding β-actin were shown and IBA1 / β-actin *ratios* were calculated. The means of IBA1 levels were represented for all groups (n = 14, 15 and 15 for PEG, Resv and Vini group, respectively). The results were expressed as percentage of PEG-treated mice as controls (rounds represent females, triangles represent males). The dotted line represents 100% signal. **Panel C:** Cropped blots of GFAP and corresponding β-actin were shown and GFAP / β-actin *ratios* were calculated. The means of GFAP levels were represented for all groups (n = 13, 14 and 13 for PEG, Resv and Vini, respectively). The results were expressed as percentage of PEG-treated mice as controls (rounds represent females, triangles represent males). The dotted line represents 100% signal. Full-length blots are presented in Supplementary Figure 3.
