# Supplementary material for: Trans ε-Viniferin Decreases Amyloid Deposits With Greater Efficiency Than Resveratrol in an Alzheimer’s Mouse Model
Source: Front Neurosci. 2022 Jan 6;15:803927. doi: 10.3389/fnins.2021.803927 (PMC8770934; doi:10.3389/fnins.2021.803927)
Supplement: Supplementary file 4 [file Image_4.pdf]

## Supplementary Figure 4

## Original blots of Figure 2C

### Blot WT IBA1

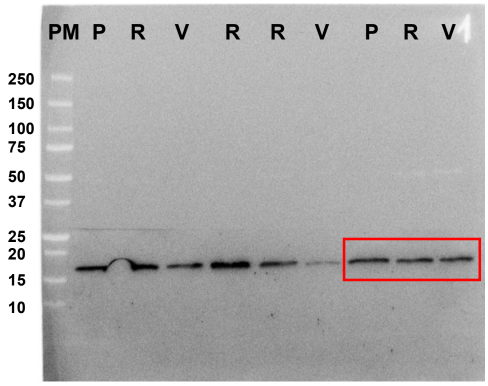


### Blot WT β-Actin

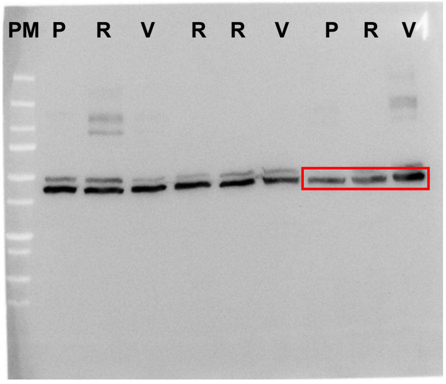


### Blot AD IBA1

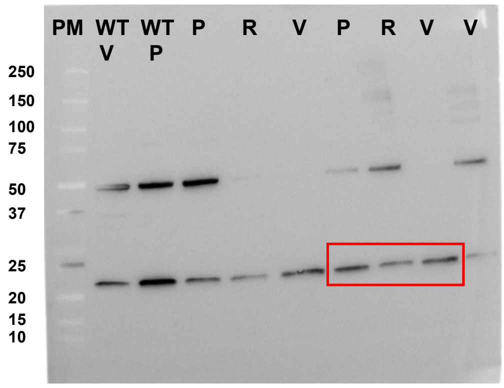


### Blot AD β-Actin

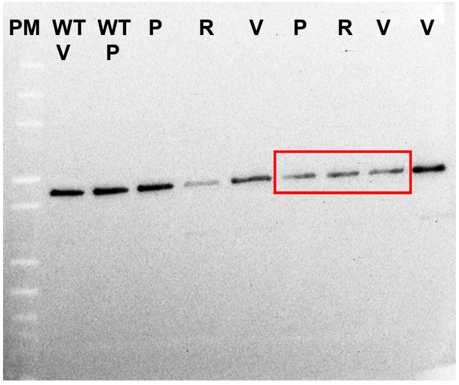


PM: kDa

WT: Wild-type (other wells loaded with AD samples)

P: PEG treatment

R: Resveratrol treatment

V: Viniferin treatment

**Original blots of Figure 3B**

**Blot WT GFAP**

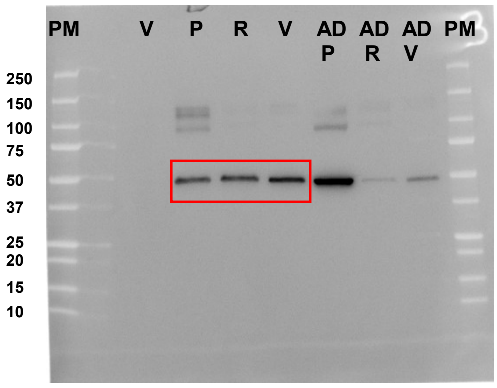


**Blot WT β-Actin**

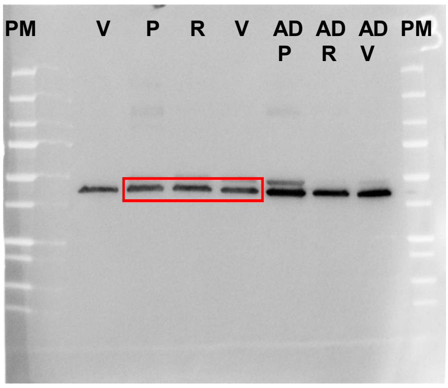


AD: Alzheimer's disease (other wells loaded with WT samples)

**Blot AD GFAP**

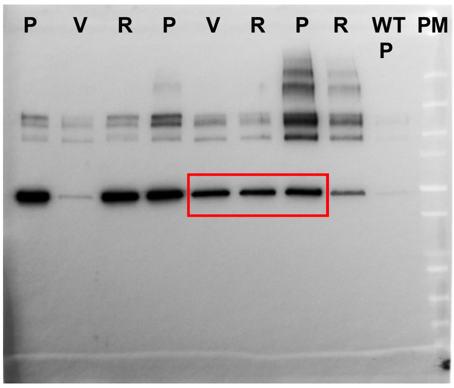


**Blot AD β-Actin**

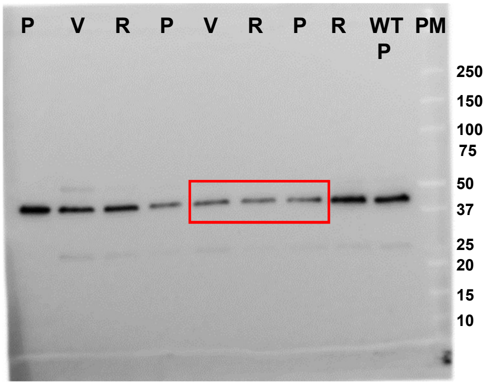


PM: kDa

WT: Wild-type (other wells loaded with AD samples)

P: PEG treatment

R: Resveratrol treatment

V: Viniferin treatment

## Original blots of Supplementary Figure 2B

**Blot IBA1**

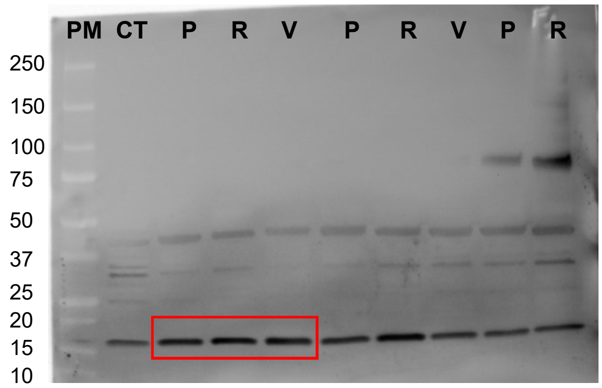


**Blot β-Actin**

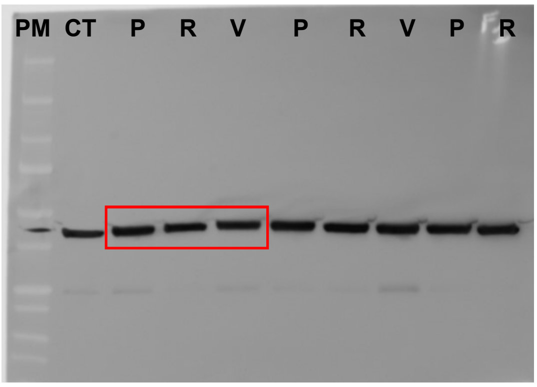


PM: kDa

CT: positive control

P: PEG treatment

R: Resveratrol treatment

V: Viniferin treatment

## Original blots of Supplementary Figure 2C

**Blot GFAP**

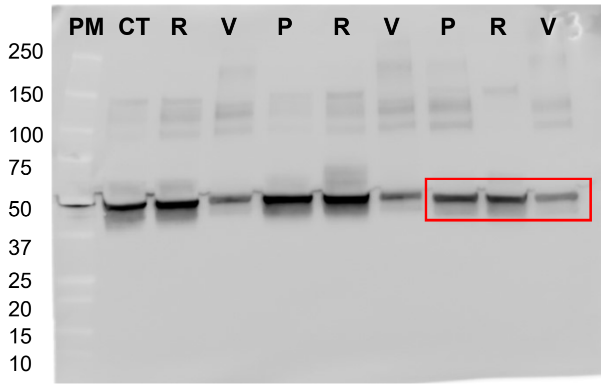


**Blot β-Actin**

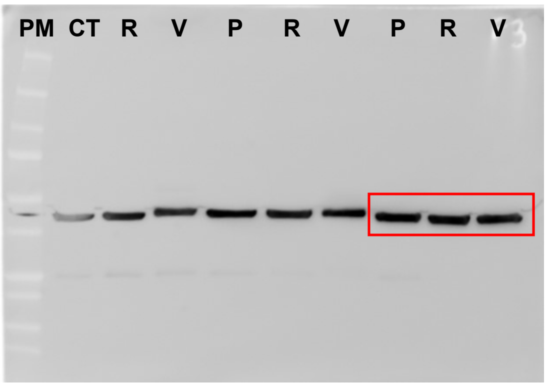


PM: kDa

CT: positive control

P: PEG treatment

R: Resveratrol treatment

V: Viniferin treatment
