# Supplementary material for: Trans ε-Viniferin Decreases Amyloid Deposits With Greater Efficiency Than Resveratrol in an Alzheimer’s Mouse Model
Source: Front Neurosci. 2022 Jan 6;15:803927. doi: 10.3389/fnins.2021.803927 (PMC8770934; doi:10.3389/fnins.2021.803927)
Supplement: Supplementary file 5 [file Table_1.pdf]

Supplementary Table 1: Chemical products used in the current study.

Chemical product	Company
Sodium fluoride (NaF), Phenylmethylsulfonyl fluoride (PMSF), Protease and phosphatase inhibitor cocktails, Dithiothreitol (DTT), Paraformaldehyde (PFA), 4',6-diamidino-2-phenylindole (DAPI), all reagent-grade chemicals for buffers	Sigma (St Quentin Fallavier, France)
Ketamidor® (Ketamine) and 2% Rompun® (Xylazine)	Coveto (Montaigu, France)
Protease-Free Bovine Serum Albumin (BSA)	Jackson ImmunoResearch Europe Ltd (Interchim distributor, France)
Laemmli Sample Buffer, 4-20% Tris-Glycine gels, Tris-Glycine SDS Running Buffer, Precision plus protein™ standards All blue, Protein Assay Dye Reagent Concentrate	Bio-rad (Marnes-la Coquette, France)
Amyloid ELISA kit	Gibco-Invitrogen (Fisher Bioblock Scientific distributor, France)
Guanidine	Acros Organics (USA)
Absolute ethanol	Carlo Erba Reagents Rodano (France)
Histosol plus	Shandon (France)
Trans ε-viniferin and *trans*-resveratrol	Locally extracted and purified
Mouse antibody anti amyloid peptide (clone W02), Chemiluminescence Luminata Forte and Classico Substrates	Millipore (Saint-Quentin-en- Yvelines, France)
Rabbit antibody anti GFAP, Donkey antibody anti goat IgG-HRP, Goat antibody anti rabbit IgG-HRP, Horse antibody anti mouse IgG-HRP	Cell Signaling Technology (Ozyme, France))
Goat antibody anti IBA-1	Abcam (France)
Mouse antibody anti β-actin	Sigma Aldrich (Merck-Millipore, France)
Donkey antibodies anti-mouse-Alexa 488, anti-rabbit-RRX, anti-goat RRX	Jackson ImmunoResearch Europe Ltd (Interchim distributor, France)
