# Supplementary material for: Trans ε-Viniferin Decreases Amyloid Deposits With Greater Efficiency Than Resveratrol in an Alzheimer’s Mouse Model
Source: Front Neurosci. 2022 Jan 6;15:803927. doi: 10.3389/fnins.2021.803927 (PMC8770934; doi:10.3389/fnins.2021.803927)
Supplement: Supplementary file 6 [file Table_2.pdf]

Supplementary Table 2: Primary and secondary antibodies used in the study.

Primary antibodies			
**Antibodies**	**Dilution for WB**	**Dilution for IF**	**Sources**
Mouse anti amyloid peptide (clone W02)	/	1:100	Millipore
Rabbit anti GFAP	1:1000	1:100	Cell signaling
Goat anti IBA-1	1:500	1:100	Abcam
Mouse anti β-actin	1:10000	/	Sigma Aldrich
Secondary antibodies			
**Antibodies**	**Dilution for WB**	**Dilution for IF**	**Sources**
Donkey anti-mouse-Alexa 488	/	1:50	Jackson immunoresearch
Donkey anti-rabbit-RRX	/	1:50	Jackson immunoresearch
Donkey anti-goat RRX	/	1:50	Jackson immunoresearch
Donkey anti goat IgG-HRP	1:1000	/	Santa cruz
Goat anti rabbit IgG-HRP	1:1000	/	Cell Signaling Technology
Horse anti mouse IgG-HRP	1:1000	/	Cell Signaling Technology

Primary antibodies			
**Antibodies**	**Dilution for WB**	**Dilution for IF**	**Sources**
Mouse anti amyloid peptide (clone W02)	/	1:100	Millipore
Rabbit anti GFAP	1:1000	1:100	Cell signaling
Goat anti IBA-1	1:500	1:100	Abcam
Mouse anti β-actin	1:10000	/	Sigma Aldrich
Secondary antibodies			
**Antibodies**	**Dilution for WB**	**Dilution for IF**	**Sources**
Donkey anti-mouse-Alexa 488	/	1:50	Jackson immunoresearch
Donkey anti-rabbit-RRX	/	1:50	Jackson immunoresearch
Donkey anti-goat RRX	/	1:50	Jackson immunoresearch
Donkey anti goat IgG-HRP	1:1000	/	Santa cruz
Goat anti rabbit IgG-HRP	1:1000	/	Cell Signaling Technology
Horse anti mouse IgG-HRP	1:1000	/	Cell Signaling Technology
